# Identification of Acupoint Indication from Reverse Inference: Data Mining of Randomized Controlled Clinical Trials

**DOI:** 10.3390/jcm9093027

**Published:** 2020-09-20

**Authors:** Ye-Chae Hwang, In-Seon Lee, Yeonhee Ryu, Ye-Seul Lee, Younbyoung Chae

**Affiliations:** 1 Acupuncture & Meridian Science Research Center, College of Korean Medicine, Kyung Hee University, Seoul 02447, Korea; mma3206@khu.ac.kr (Y.-C.H.); inseon.lee@khu.ac.kr (I.-S.L.); 2 KM Fundamental Research Division, Korea Institute of Oriental Medicine, Daejeon 34054, Korea; yhryu@kiom.re.kr; 3 Department of Anatomy and Acupoint, College of Korean Medicine, Gachon University, Seongnam 13306, Korea

**Keywords:** acupoint indication, acupoint specificity, Bayesian modeling, data mining, clinical trials

## Abstract

The specificity of acupoint indication (i.e., reverse inference—diseases for which an acupoint could be used) might differ from the specificity of acupoint selection (i.e., forward inference—acupoints used for a disease). In this study, we explore acupoint specificity through reverse inferences from the dataset of prescribed acupoints for a certain disease in clinical trials. We searched acupuncture treatment regimens in randomized controlled trials included in the Cochrane Database of Systematic Reviews. For forward inference, the acupoints prescribed for each disease were quantified. For reverse inference, diseases for each acupoint were quantified. Data were normalized using Z-scores. Bayes factor correction was performed to adjust for the prior probability of diseases. The specificities of acupoint selections in 30 diseases were determined using forward inference. The specificities of acupoint indications regarding 49 acupoints were identified using reverse inference and then subjected to Bayes factor correction. Two types of acupoint indications were identified for 24 acupoints: regional and distal. Our approach suggests that the specificity of acupoint indication can be inferred from clinical data using reverse inference. Acupoint indication will improve our understanding of acupoint specificity and will lead to the establishment of a new model of analysis and educational resources for acupoint characteristics.

## 1. Introduction

The action of acupuncture can be explained by various levels of neuromodulations by means of local, segmental, and general effects (descending analgesia and central regulation) [1,2]. Many neuroimaging studies have shown common brain activations and deactivation patterns following acupuncture stimulations [3]. However, it has been difficult to reveal the point specificities on clinical significances using these approaches [4,5]. Clinical acupoint selection involves three basic principles: (1) local acupoints near the area where symptoms occur, (2) distant acupoints from the symptom location along the meridian, and (3) distant acupoints based on symptom differentiation, regardless of symptom location [6,7,8]. Recent studies have investigated these principles through the novel paradigm of data mining, using a number of available datasets related to traditional medicine. The associations between symptom differentiations and acupuncture prescriptions are relatively clear in classical textbooks, although the associations from these classical textbooks are not likely to be prominent in clinical practice [9,10]. Previous studies have identified selections of acupoints for various diseases such as lumbar disc herniation, dysmenorrhea, rheumatoid arthritis, and visceral pain [11,12,13,14]. However, these findings do not directly reveal the specificity of acupoint indication; they only indicate which acupoints can be selected specifically for a particular disease [15].

In clinical practice, it is essential to identify which acupoints are specifically associated with a particular disease. However, selecting an acupoint to treat a particular disease does not always imply that the selected acupoint has specific indications for that disease [15]. Thus, indications for the selected acupoints may not always match the target disease for which the acupoints were selected. This crucial point is often missed in both education and clinical practice, which often leads to errors in logic. To clarify this relationship, we suggested two-directional relationships between diseases and acupoints: forward inference and reverse inference. Forward inference analysis identifies the specificity of acupoint selection by analyzing the probability that an acupoint would be selected for a given disease: P (Acupoint | Disease). In contrast, reverse inference analysis identifies the specificity of acupoint indication by analyzing the probability of a given disease based on the selected acupoint: P (Disease | Acupoint). In our previous study, we proposed that the specificity of acupoint selection might differ from the specificity of acupoint indication. For example, BL23, GB30, and GV3 showed strong forward inferences to lumbar herniated intervertebral disc, while lumbar herniated intervertebral disc showed a high reverse inference score for acupoint BL23 [15].

Implementation of forward and reverse inferences, based on a large database, is needed to clarify the associations between diseases and acupoints. The Cochrane Database of Systematic Reviews (CDSR) identifies, appraises, and synthesizes all empirical evidence that meets prespecified eligibility criteria for efficacy [16]. Therefore, we hypothesized that we could identify the specificity of acupoint indication by analyzing relationships between prescribed acupoints and diseases using CDSR data. We also applied data mining techniques and Bayes factor (BF) correction to CDSR data. Data mining can reveal associations between diseases and acupoints [1,15]. Bayes factor correction has been suggested as a way to avoid logical errors and fallacies in forward and reverse inference analyses [17,18,19,20]. Bayes factor correction can help define the specificities of acupoint indications by correcting the odds of the prior probability or the odds of the diseases presented in the data. By eliminating the prior probability of diseases, the substance of reverse inference can be formalized to determine the specificities of acupoint indications.

In the current study, we searched acupuncture regimens in randomized controlled trials included in the CDSR and then identified the specificities of acupoint selections and acupoint indications. We explored the specificity of acupoint selections using forward inference and the specificity of acupoint indications using reverse inference based on clinical trial data.

## 2. Methods

### 2.1. Data Extraction Process

**Figure 1:**
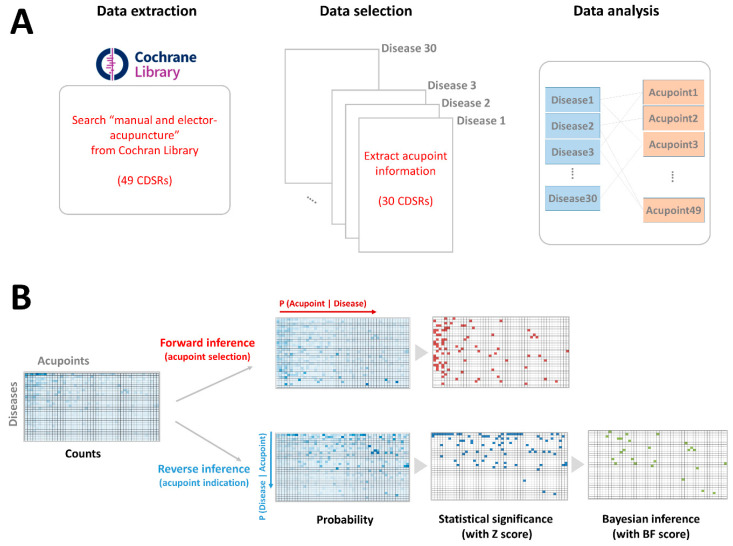
Data extraction and data analysis. (**A**): Data extraction procedures. The frequencies of the co-occurrences of the 49 acupoints and 30 diseases were extracted to explore the associations between diseases and acupoints. (**B**): Data analysis procedures. For forward inference, acupoints prescribed for each disease were quantified. For reverse inference, diseases for each acupoint were quantified. Probability values were Z-transformed to assess statistical significance for both forward and reverse inference (Z > 1.96). Bayes factor correction was applied to reverse inference to adjust for the odds of the prior probability of the disease. BF: Bayes factor

To extract acupoint information from randomized controlled clinical trials, we searched the Cochrane Library (www.cochranelibrary.com) using the keyword “acupuncture” in records titled Cochrane Reviews. To ensure a sufficient number of studies of each disease in the frequency analysis, studies involving more than three clinical trials with acupoint information were included. From the retrieved studies, the following features were included for analysis: all relevant diseases, interventions using needle-type acupuncture (manual and electroacupuncture), and all acupuncture regimens (both standardized and semistandardized). Studies were excluded from analysis if they involved interventions with non-needle type acupuncture (e.g., acupressure and laser acupuncture), interventions that were not acupuncture (e.g., moxibustion), or acupuncture trials using only a subset of body areas for treatment (e.g., ears, face, head, or feet). Therefore, a total of 49 CDSR records were retrieved from the Cochran Library, and 30 CDSR records met for our criteria. A total of 421 randomized clinical trials were included for this analysis of a total of 30 diseases (i.e., 63 studies for depression, 30 studies for dysmenorrhea, and 25 studies for lower back pain). To remove possible outliers and include only acupoints with sufficient numbers of prescriptions, acupoints were included if they were used more than 14 times across all studies, for a total of 49 acupoints. To explore the associations between diseases and acupoints, the frequencies of co-occurrences of the 49 acupoints and 30 diseases were extracted (Figure 1).

### 2.2. Data Analysis of Acupoint Selection Using Forward Inference

The specificity of acupoint selection can be inferred from observed data, with the probability of selecting one acupoint for a certain disease represented as P (Acupoint | Disease). The acupoint selection specificity was defined by forward inference (the probability that an acupoint would be selected for a given disease), calculated as the frequency of selection of that acupoint divided by the total number of acupoint selections. Probability values were Z-transformed for assessment of statistical significance (Z > 1.96).

### 2.3. Data Analysis of Acupoint Indication Using Reverse Inference

The specificity of an acupoint indication can be inferred from the observed data, with the probability of disease targeted by a certain acupoint represented as P (Disease | Acupoint). The acupoint indication specificity was defined by reverse inference (i.e., Bayesian inference, the probability that a given disease would be defined as the indication for a certain acupoint), calculated as the frequency selection of that disease divided by the total number of disease selections. Probability values were Z-transformed to assess statistical significance (Z > 1.96).

### 2.4. Bayes Factor Correction of Acupoint Indication

While conditional probability used in reverse inference helps clarify the probability of diseases in acupoint indications, it does not consider the prior odds of the diseases. To consider the prior probability of the diseases, Bayes factor correction was applied to the reverse inference results. According to Bayes’ theorem, BF is the likelihood ratio of the marginal likelihood of one particular hypothesis to another. We investigated the relative strength of each hypothesis compared with the null hypothesis (e.g., $H_{\alpha }$ = disease K is highly treated by acupoint L in randomized controlled trials) by assessing BF (*θ* = data) as below (1).

(1) BFαN= p(θ|Hα)p(θ|HN)=p(Dk|Al)1−p(Dk|Al)p(Dk)1−p(Dk)                    

To determine BF, the odds for the presence of disease when one acupoint is used were qualified by the odds of the prior probability of the disease (i.e., number of acupoints used for disease, the denominator). BF > 3 indicated substantial evidence in favor of the inference.

## 3. Results

### 3.1. Patterns of Acupoint Selection for 30 Diseases Using Forward Inference

**Figure 2:**
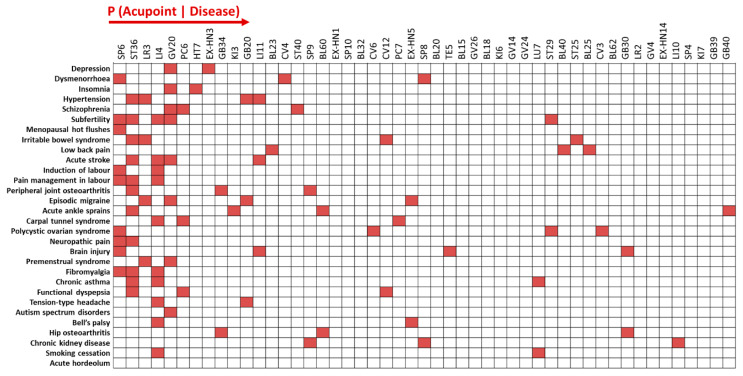
Patterns of acupoint selection for 30 diseases using forward inference. The most frequently selected acupoints were similar across the 30 diseases, while different patterns of selected acupoints were found for each disease. Z-scores indicate the frequency of each acupoint in each disease trial, compared to the overall acupoint selection pattern for that disease.

Figure 2 lists the 49 most frequently prescribed acupoints in descending order of their probability values (*p*) for the 30 diseases. Among all diseases, the most frequently prescribed acupoints were SP6 (*p* = 0.082), ST36 (*p* = 0.068), LR3 (*p* = 0.066), and LI4 (*p* = 0.064). The most frequently selected acupoints were similar across the 30 diseases, whereas different patterns of selected acupoints were found for each disease.

The Z-scores illustrate how frequently each acupoint was used in each disease trial compared to the overall acupoint selection pattern for that disease. The Z-scores of the 49 acupoints for the 30 diseases are depicted on a heatmap. For example, acupoints with a high Z-score (Z > 1.96) for the 3 main diseases are as follows: GV20 (Z = 3.82) and EX-HN3 (Z = 2.65) for depression; SP6 (Z = 4.29), CV4 (Z = 2.37), and SP8 (Z = 2.37) for dysmenorrhea; HT7 (Z = 3.55) and GV20 (Z = 1.98) for insomnia (Figure 2).

### 3.2. Patterns of Acupoint Indications for 49 Acupoints Using Reverse Inference

**Figure 3:**
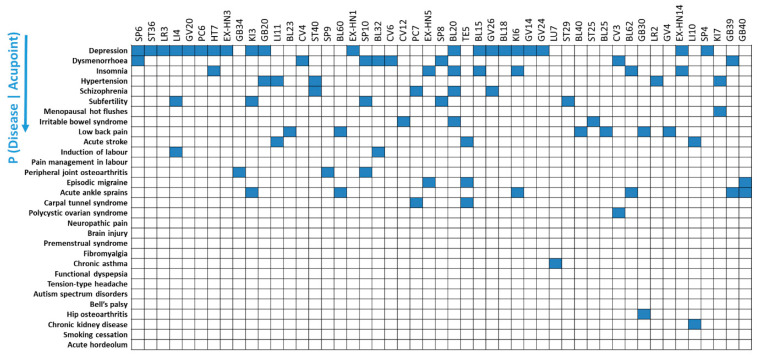
Patterns of acupoint indications for 49 acupoints using reverse inference. Depression was the most prominent indication across the 49 acupoints, while different acupoint indications were also found for each acupoint. Z-scores indicate the frequency at which each disease was treated by each acupoint compared to the indications for that acupoint.

Figure 3 lists the 30 diseases in descending order of their overall trial frequencies for the 49 acupoints. Depression was the most prominent acupoint indication across the 49 acupoints, although different acupoint indications were also found for each acupoint.

The Z-scores illustrate how frequently each disease was treated by each acupoint, compared to the indications for that acupoint. The Z-scores of the 30 indications for the 49 acupoints are depicted on a heatmap. For example, diseases with a high Z-score (Z > 1.96) for the 4 main acupoints are as follows: depression (Z = 3.28) and dysmenorrhea (Z = 2.54) for SP6; depression (Z = 2.71) for ST36; depression (Z = 4.07) for LR3; depression (Z = 3.13), induction of labor (Z = 2.26), and subfertility (Z = 2.26) for LI4 (Figure 3).

### 3.3. Specificity of Acupoint Indications Using Bayes Factor Correction

**Figure 4:**
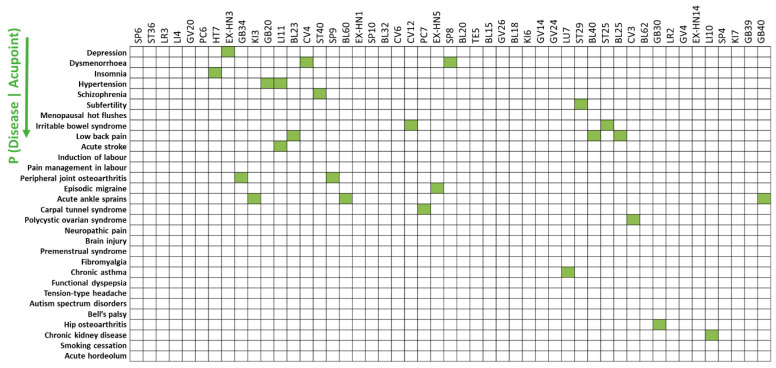
Specificity of acupoint indications using Bayes factor (BF) correction. The acupoint indications were more specific to the respective diseases after BF correction (BF > 3).

The acupoint indications were more specific to each disease after correction with BF (BF > 3). Diseases with a high BF score (BF > 3) for acupoints are as follows: insomnia (BF = 3.69) for HT7; depression (BF = 6.36) for EX-HN3; peripheral joint osteoarthritis (BF = 7.60) for GB34; acute ankle sprains (BF = 5.39) for KI3 (Figure 4). The results indicate that few acupoint indications for diseases with greater prior probabilities, such as depression and dysmenorrhea, did not survive the BF correction.

### 3.4. Two Different Patterns of Acupoint Indications

**Figure 5:**
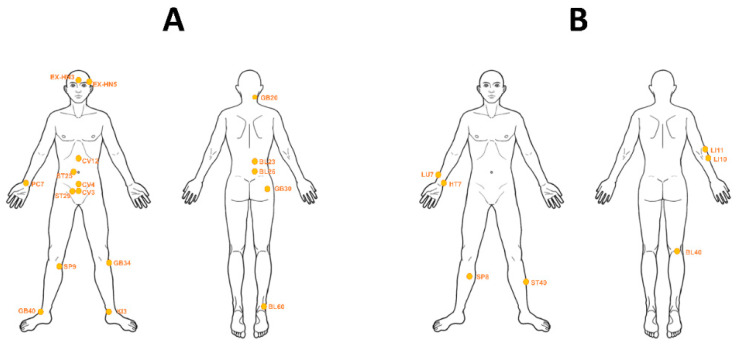
Two patterns of acupoint indications. (**A**) Type 1 includes acupoints with regional indications distributed in the head, back, abdomen, and ankle (*n* = 17; BL23, BL25, BL60, CV3, CV4, CV12, EX-HN3, EX-HN5, GB20, GB30, GB34, GB40, KI3, PC7, SP9, ST25, ST29). (**B**) Type 2 includes acupoints with distal indications distributed in the upper/lower limbs (*n* = 7; BL40, HT7, LI10, LI11, LU7, SP8, ST40).

After BF correction, the acupoint indications were classified into two categories based on the distance between acupoint and disease area. Acupoints with regional indications were distributed in the head, back, abdomen, and ankle (type 1, *n* = 17). Acupoints with distal indications were mainly distributed in the upper/lower limbs (type 2, *n* = 7; Table 1). The acupoints were overlaid on a human body template (Figure 5).

## 4. Discussion

The findings of the present study revealed patterns of acupoint selection for 30 diseases, as well as patterns of acupoint indications for 49 acupoints, using clinical trial data from the CDSR. We demonstrated the specificity of acupoint selection for each disease using forward inference. We also demonstrated the specificity of acupoint indication using reverse inference. Considering the prior probabilities of diseases, acupoint indications were further refined with BF correction. Finally, we identified two types of acupoint indications for 24 acupoints: regional and distal. Identification of the specific patterns of acupoint indications through data mining from clinical trials will be useful for understanding the characteristics of acupoints in education and clinical practice.

The core set of acupoints can be widely used to treat a variety of diseases, whereas some acupoints are only used to treat specific diseases. In our previous study, we identified the commonality and specificity of acupoint selections based on virtual acupuncture treatments prescribed by practicing clinicians. We found that acupoints ST36, LI4, and LR3 were the most commonly prescribed across all diseases [21]. Mining of CDSR data also revealed that the main acupoints commonly used for a variety of pain management approaches were SP6, ST36, LI4, and LR3 [1]. Consistent with the findings of previous studies, we found that the core acupoints were widely used for all 30 diseases, regardless of disease type. In contrast, acupoints GV20 and EX-HN3 were specifically used for the treatment of depression, while acupoints CV4 and SP8 were specifically used for the treatment of dysmenorrhea (Figure 2). These findings support the common rule of acupoint selections, in which some acupoints are common among all diseases while other acupoints are specifically prescribed for the treatment of certain diseases.

Forward and reverse inference have been used in various areas of academic research. Notably, reverse inference has been used in neuroscience to assign a certain cognitive process to activation of a certain brain region, while forward inference has been used to evaluate cognitive theories based on different patterns of brain activation [18,20]. As forward and reverse inferences can link associations directly, they demonstrate causal relationships without possible logical errors. This study extends the concept of forward and reverse inference in terms of the relationship between acupoints and diseases using CDSR data. The specificity of acupoint indication is generally inferred from the specificity of acupoint selection. However, we demonstrated that forward inference alone can lead to errors in logic regarding the acupoint indication specificity and that the application of reverse inference can improve the acupoint specificity.

Previous studies have indicated that reverse inference should be approached with caution if the prior probability (i.e., the odds of the prior probability of the disease) remains unknown [19]. By implementing reverse inference, we found that depression and dysmenorrhea were specific indications for acupoint SP6, while depression, induction of labor, and subfertility were specific indications for acupoint LI4 (Figure 3). However, the total number of trials can influence the specificity of acupoint indications. For example, depression was an indication for many acupoints (e.g., SP6, ST36, LR3, LI4) in the reverse inference because the total number of studies on depression was greater than the number of studies on other diseases. To avoid possible fallacy following the distortion of data, BF correction was implemented when identifying the specificity of acupoint indications; this approach corrected the odds of the prior probability of the disease. Implementation of BF correction led to the identification of insomnia as the indication for acupoint HT7 while ruling out diseases with high frequency in the data (e.g., depression) and specific acupoints (e.g., EX-HN3) (Figure 4). This analysis demonstrated that reverse inference using BF correction is useful for inferring specificity of acupoint indications.

In the current study, two types of acupoint indications were identified: regional and distal. Both regional and distal acupoints were specifically linked with certain diseases. Among regional acupoints, low back pain was the indication for acupoint BL23 in the back, while acute ankle sprain was the indication for acupoint BL60 in the feet. Among distal acupoints, low back pain was the indication for BL40 in the lower limb, while insomnia was the indication for acupoint HT7 in the wrist (Figure 5). Classification of acupoint indications into regional and distal types is consistent with the approach used in previous studies, where both regional and distal acupoints were mainly used for pain management during acupuncture, based on data mining from clinical trials [1,8,11].

Two different strategies for acupoint prescription, including branch treatment and root treatment, are widely used in the context of traditional East Asian medicine [22]. Pattern identification can extract and synthesize patients’ signs and symptoms and lead to making treatment decisions [23]. Acupuncture practitioners can choose the most appropriate acupoints based on the disease or symptoms (i.e., branch treatment) or on the results of pattern identification (i.e., root treatment) [21,24]. Although some clinical trials consider individualized treatment strategies, clinical trials are generally conducted to find out the efficacy of the acupuncture treatment on a certain disease. Furthermore, pattern identification can be varied across patients even though they have the same disease [25]. Only a limited number of studies provided the information of pattern identification from the CDSR. Thus, the present study only determined the acupoint indication based on the relationship between disease/symptoms and acupoints without considering pattern identification. The acupoint indication associated with pattern identification should be further studied in the future.

Our study had several limitations. First, it did not consider the effectiveness of the acupuncture treatment in each study. To include the effectiveness of acupoints, future studies with appropriate control groups will be needed to enable the extraction of the effectiveness of acupuncture treatment administered to a group of acupoints. Due to the limited number of included studies, we were not able to analyze the data considering the clinical effectiveness of needling at each point. More sufficient data will be needed to ensure the acupoint indications based on clinical significance in the future. Second, this study did not restrict methodological quality among the assessed studies. Insufficient evidence was available to reveal discrepancies between acupoints used in high-quality and low-quality studies. Future analyses should incorporate inclusion criteria to ensure the inclusion of studies with meaningful information. Finally, this study identified acupoint indications only from clinical trials. Additional analyses will be needed to clarify the associations between diseases and acupoints based on clinical data obtained in a real-world setting.

In summary, data mining, forward and reverse inference analyses, and BF correction of clinical trial data revealed the bidirectional specificity of acupoints for various diseases. We expect our approach to provide new information regarding the specificities of acupoint indications from clinical observations.

## Figures and Tables

**Table 1:** Classification of acupoint indications with strong inference (BF > 3).

**Type I (Regional Acupoints, *n* = 17)**	
BL23 (back)	Low back pain (BF = 10.12)
BL25 (back)	Low back pain (BF = 36.87)
BL60 (feet)	Acute ankle sprain (BF = 13.47)
CV3 (abdomen)	Polycystic ovarian syndrome (BF = 18.53)
CV4 (abdomen)	Dysmenorrhea (BF = 5.51)
CV12 (abdomen)	Irritable bowel syndrome (BF = 8.29)
EX-HN3 (head)	Depression (BF = 6.36)
EX-HN5 (head)	Episodic migraine (BF = 9.03)
GB20 (head)	Hypertension (BF = 3.94)
GB30 (lower limb)	Hip osteoarthritis (BF = 53.94)
GB34 (lower limb)	Peripheral joint osteoarthritis (BF = 7.60)
GB40 (feet)	Acute ankle sprain (BF = 38.50)
KI3 (feet)	Acute ankle sprain (BF = 5.39)
PC7 (upper limb)	Carpal tunnel syndrome (BF = 35.31)
SP9 (lower limb)	Peripheral joint osteoarthritis (BF = 9.39)
ST25 (abdomen)	Irritable bowel syndrome (BF = 33.16)
ST29 (abdomen)	Subfertility (BF = 27.09)
**Type II (Distal Acupoints, *n* = 7)**	
BL40 (lower limb)	Low back pain (BF = 32.26)
HT7 (upper limb)	Insomnia (BF = 3.68)
LI10 (upper limb)	Chronic kidney disease (BF = 105.3)
LI11 (upper limb)	Hypertension (BF = 6.60), Acute stroke (BF = 6.57)
LU7 (upper limb)	Chronic asthma (BF = 22.48)
SP8 (lower limb)	Dysmenorrhea (BF = 17.56)
ST40 (lower limb)	Schizophrenia (BF = 5.29)

## References

[1] Hwang Y.C., Lee I.S., Ryu Y., Lee M.S., Chae Y. (2020). Exploring traditional acupuncture point selection patterns for pain control: Data mining of randomised controlled clinical trials. Acupunct. Med..

[2] White A. (2009). Editorial Board of Acupuncture in M. Western medical acupuncture: A definition. Acupunct. Med..

[3] Chae Y., Chang D.S., Lee S.H., Jung W.M., Lee I.S., Jackson S., Kong J., Lee H., Park H.J., Lee H. (2013). Inserting needles into the body: A meta-analysis of brain activity associated with acupuncture needle stimulation. J. Pain.

[4] Jung W.M., Lee I.S., Lee Y.S., Kim J., Park H.J., Wallraven C., Chae Y. (2019). Decoding spatial location of perceived pain to acupuncture needle using multivoxel pattern analysis. Mol. Pain.

[5] Lee I.S., Jung W.M., Park H.J., Chae Y. (2020). Spatial Information of Somatosensory Stimuli in the Brain: Multivariate Pattern Analysis of Functional Magnetic Resonance Imaging Data. Neural Plast..

[6] Chen Y.R., Zhu J., Song J.S., She Y.F. (2012). Discussion on point selection and compatibility of acupuncture formula. Zhongguo Zhen Jiu.

[7] Jung W.M., Lee T., Lee I.S., Kim S., Jang H., Kim S.Y., Park H.J., Chae Y. (2015). Spatial Patterns of the Indications of Acupoints Using Data Mining in Classic Medical Text: A Possible Visualization of the Meridian System. Evid. Based Complement. Altern. Med..

[8] Lee S.H., Kim C.E., Lee I.S., Jung W.M., Kim H.G., Jang H., Kim S.J., Lee H., Park H.J., Chae Y. (2013). Network analysis of acupuncture points used in the treatment of low back pain. Evid. Based Complement. Altern. Med..

[9] Alvim D.T., Ferreira A.S. (2018). Inter-expert agreement and similarity analysis of traditional diagnoses and acupuncture prescriptions in textbook- and pragmatic-based practices. Complement. Ther. Clin. Pract..

[10] Lee T., Jung W.M., Lee I.S., Lee Y.S., Lee H., Park H.J., Kim N., Chae Y. (2014). Data Mining of Acupoint Characteristics from the Classical Medical Text: DongUiBoGam of Korean Medicine. Evid. Based Complement. Altern. Med..

[11] Lee I.S., Cheon S., Park J.Y. (2019). Central and Peripheral Mechanism of Acupuncture Analgesia on Visceral Pain: A Systematic Review. Evid. Based Complement. Altern. Med..

[12] Li J.B., Xiong Q.L., Qu S.K., Qi F., Zhang L., Wang Q., Bao K., Li F.B. (2013). Discussion on the regular of acupoint selection of acupuncture and moxibustion for lumbar disc herniation during recent 10 years. Zhongguo Zhen Jiu.

[13] Yu S., Yang J., Ren Y., Chen L., Liang F., Hu Y. (2015). Characteristics of acupoints selection of moxibustion for primary dysmenorrhea based on data mining technology. Zhongguo Zhen Jiu.

[14] Zhang K., Xu Y., Ding S., Hong S., Zhao X., Guo Y. (2017). Literature study for acupoint selection rule of rheumatoid arthritis treated with acupuncture. Zhongguo Zhen Jiu.

[15] Hwang Y.C., Lee Y.S., Ryu Y., Lee I.S., Chae Y. (2020). Statistical inference of acupoint specificity: Forward and reverse inference. Integr. Med. Res..

[16] Smith C.A., Armour M., Lee M.S., Wang L.Q., Hay P.J. (2018). Acupuncture for depression. Cochrane Database Syst Rev..

[17] Heit E. (2014). Brain imaging, forward inference, and theories of reasoning. Front. Hum. Neurosci..

[18] Henson R. (2006). Forward inference using functional neuroimaging: Dissociations versus associations. Trends Cogn Sci..

[19] Hutzler F. (2014). Reverse inference is not a fallacy per se: Cognitive processes Can be inferred from functional imaging data. Neuroimage.

[20] Poldrack R.A. (2006). Can cognitive processes be inferred from neuroimaging data?. Trends Cogn Sci..

[21] Lee Y.S., Ryu Y., Yoon D.-E., Kim C.H., Hong G., Hwang Y.C., Chae Y. (2020). Commonality and specificity of acupuncture points selections. Evid. Based Complement. Altern. Med..

[22] Lee Y.S., Ryu Y., Chae Y. (2020). Acupoint selection based on pattern identification results or disease state. Integr. Med. Res..

[23] Birch S., Alraek T. (2014). Traditional East Asian medicine: How to understand and approach diagnostic findings and patterns in a modern scientific framework?. Chin. J. Integr. Med..

[24] Kim C.H., Yoon D.E., Lee Y.S., Jung W., Kim J.H., Chae Y. (2019). Revealing Associations between Diagnosis Patterns and Acupoint Prescriptions Using Medical Data Extracted from Case Reports. J. Clin. Med..

[25] Kam W., Zhang Z.J., Baarnhielm S. (2019). Traditional Chinese Medicine Explanatory Models of Depressive Disorders: A Qualitative Study. Cult. Med. Psychiatry.

